# SNP-PHAGE – High throughput SNP discovery pipeline

**DOI:** 10.1186/1471-2105-7-468

**Published:** 2006-10-23

**Authors:** Lakshmi K Matukumalli, John J Grefenstette, David L Hyten, Ik-Young Choi, Perry B Cregan, Curtis P Van Tassell

**Affiliations:** 1 US Department of Agriculture, ARS, Beltsville Agricultural Research Center, Bovine Functional Genomics Laboratory, Beltsville, MD 20705, USA; 2 Bioinformatics and Computational Biology, George Mason University, Manassas, VA 20110, USA; 3 US Department of Agriculture, ARS, Beltsville Agricultural Research Center, Soybean Genomics and Improvement Laboratory, Beltsville, MD 20705, USA

## Abstract

**Background:**

Single nucleotide polymorphisms (SNPs) as defined here are single base sequence changes or short insertion/deletions between or within individuals of a given species. As a result of their abundance and the availability of high throughput analysis technologies SNP markers have begun to replace other traditional markers such as restriction fragment length polymorphisms (RFLPs), amplified fragment length polymorphisms (AFLPs) and simple sequence repeats (SSRs or microsatellite) markers for fine mapping and association studies in several species. For SNP discovery from chromatogram data, several bioinformatics programs have to be combined to generate an analysis pipeline. Results have to be stored in a relational database to facilitate interrogation through queries or to generate data for further analyses such as determination of linkage disequilibrium and identification of common haplotypes. Although these tasks are routinely performed by several groups, an integrated open source SNP discovery pipeline that can be easily adapted by new groups interested in SNP marker development is currently unavailable.

**Results:**

We developed SNP-PHAGE (**SNP **discovery **P**ipeline with additional features for identification of common haplotypes within a sequence tagged site (**H**aplotype **A**nalysis) and **Ge**nBank (-dbSNP) submissions. This tool was applied for analyzing sequence traces from diverse soybean genotypes to discover over 10,000 SNPs. This package was developed on UNIX/Linux platform, written in Perl and uses a MySQL database. Scripts to generate a user-friendly web interface are also provided with common queries for preliminary data analysis. A machine learning tool developed by this group for increasing the efficiency of SNP discovery is integrated as a part of this package as an optional feature. The SNP-PHAGE package is being made available open source at .

**Conclusion:**

SNP-PHAGE provides a bioinformatics solution for high throughput SNP discovery, identification of common haplotypes within an amplicon, and GenBank (dbSNP) submissions. SNP selection and visualization are aided through a user-friendly web interface. This tool is useful for analyzing sequence tagged sites (STSs) of genomic sequences, and this software can serve as a starting point for groups interested in developing SNP markers.

## Background

When undertaking polymorphism discovery, the selection of an optimal tool depends on the nature of input sequences. For sequences that are derived from individual clones where there is no heterogeneity, SNP discovery can be accomplished by comparing sequence information. When relatively few reads are available, PolyBayes[1] in combination with Phrap[2] can be used. In this case, Phrap performs the sequence assembly and PolyBayes detects SNPs by implementing a Bayesian algorithm. The PolyBayes algorithm estimates the conditional probabilities accounting for Phred quality scores and depth of reads. However, for analyzing a large sequence dataset, such as shotgun sequence reads from a finished genome, ssahaSNP[3] may be optimal. The ssahaSNP software performs fast searches through custom hashing algorithm for making alignments and screens for SNP candidates using neighborhood quality standard, NQS[4]. The NQS algorithm accounts for the quality value of the bases with variation as well as the quality values in the neighboring bases. Mining of SNPs from EST sequences is an attractive proposition in some plant and animal species when genome sequences are not yet available. The steps involved in SNP discovery from EST sequences include clustering, sequence assembly and SNP detection and there are several software options available to handle each of these steps. It is important to use Phred quality scores and multiple sequence evidences in calling a putative SNP. However EST data can only provide very limited putative polymorphisms as this approach requires sequences from different genotypes and depth of reads to reduce the likelihood of false positives. Except for the most abundantly expressed genes, the numbers of redundant EST sequences are relatively low. Also other factors such as alternative splicing, reverse transcription errors, and RNA editing further interfere with the predictions even after including sequence quality scores. By constructing a software data analysis pipeline SNP discovery from EST sequences was successfully implemented for maize [5], human [6] and pine [7] species. In polyploid species like soybean SNP, discovery efforts are further complicated by polyploidy where paralog sequences have to be delineated before SNP calling can be done (manuscript in preparation).

For large-scale targeted SNP discovery from PCR amplified sequence tagged sites containing potential sequence variation(s) using a set of flanking primers, PolyPhred [8] is widely used because it can detect heterozygous bases from the two alleles within an individual. Along with the release of PolyPhred version 5.0 [9], other software such as InSNP [10], novoSNP [11] and SNPdetector [12] have recently been released. These packages are specially designed for re-sequencing projects and most of them require an anchor sequence to align individual reads to form a sequence assembly. InSNP [10] is a windows based package and can be helpful for users not familiar with Linux. SNPdetector [12] scripts work only on Unix/Linux platforms and uses the Smith-Waterman algorithm for aligning reads, as well as a modified version of the NQS [4] method for detecting homozygous SNPs among different individuals. In addition, SNPdetector requires a minimum of a 30% threshold for a secondary peak intensity for detecting heterozygous SNPs. NovoSNP [11] is designed with a graphical interface and is written in Tcl, so, it can work on windows and Unix/Linux based platforms. NovoSNP uses BLAST [13] for aligning sequence reads and uses a series of filters to reduce false positives. This package is configured to work with a database, and, hence, it makes polymorphism discovery and data storage seamless. Other polymorphism discovery software, such as autoSNP [14], that rely on redundancy and co-segregation of markers within a sequence are useful when trace data are not available.

A high-throughput polymorphism discovery project involves the analysis and management of different types of data including sequences, polymorphisms, genotypes, and haplotypes, and, hence, all of the analytical steps need to be automated and the information stored in a relational database. Bioperl [15] is a rich resource for performing several bioinformatics analyses, however, there are currently no modules available for parsing the output generated by PolyBayes and PolyPhred. The Perl package, POSA [16], contains a script for executing PhredPhrap and parsing the PolyPhred output. However, that script ignored indels, so, that program was extensively modified and used in the development of this package. The objective of the work reported here was to develop an open source package for facilitating polymorphism discovery through application of the widely used software, PolyBayes [1] and PolyPhred [8], for analysis, storing, and editing of polymorphism information in a relational database through a user friendly web interface. Additional features were desired for identifying common haplotypes within a sequence tagged site (STS) fragment and for generating data in the formats required for dbSNP submissions. Using a large annotated training dataset derived from PolyBayes predictions, our group has implemented a machine learning algorithm [17] that improved the efficiency of SNP discovery in soybean by reducing the need for expert intervention by 85%. The machine learning component is integrated as a part of SNP-PHAGE software.

## Implementation

The SNP-PHAGE software pipeline is run through a combination of UNIX/Linux command line and web browser interfaces. It was implemented in Perl and uses standard open source modules such as Bioperl [15]. SNP-PHAGE, however, requires other packages, such as Phred [18,19], Phrap [2], CrossMatch [2], PolyPhred [8], PolyBayes [1] and C4.5 [20]. All of this software is freely available for academic use from the links provided at the SNP-PHAGE website. An installation guide is provided with the SNP-PHAGE package that contains test scripts to check for prerequisite software availability. These test scripts also include test data to check the installation success, the database install, and the web interface [see Additional file 1].

SNP-PHAGE software has three components:

a) Relational database schema and tables for storing information about the STS, sample chromatograms and genotypes, consensus sequence, and polymorphisms and haplotypes;

b) Scripts for analyzing chromatograms by executing the programs described above in a given sequence (Phred, Phrap, PolyPhred and PolyBayes), parsing the program outputs for putative SNPs, and making database entries.

c) Implementation of a web interface for viewing putative SNPs; adding new SNPs; validating SNPs; and editing consensus sequence and generating a report containing confirmed SNPs. The genotype output is of the highest quality base from duplicate samples and/or sequencing reads from both forward and reverse directions. Haplotypes present in each STS are also determined.

**Figure 1:**
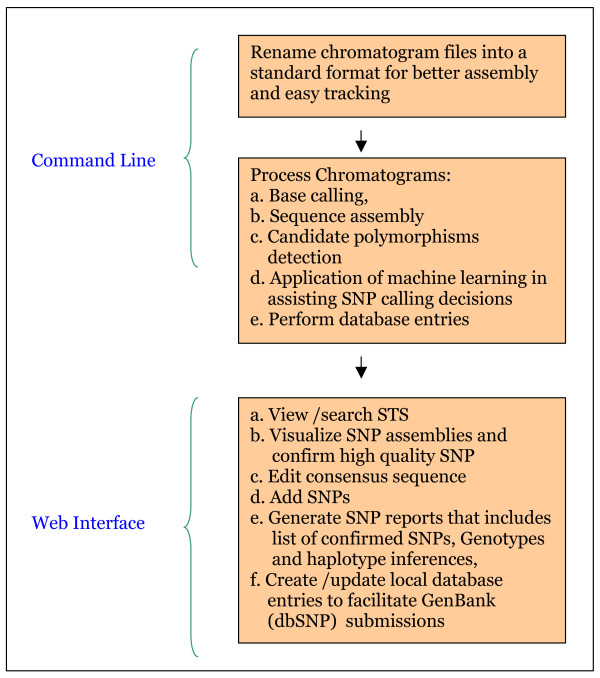
**Flow Chart of SNP-PHAGE**. Polymorphisms analysis of multiple sequence tag sites using SNP-PHAGE is effectively a three stage process where the first two stages have to be performed from a UNIX/Linux command line interface. The various tasks mentioned in the second stage are executed in sequence by running a single script. The subsequent analysis steps can be performed from a user friendly web interface.

A flow-chart containing data processing and analysis steps in SNP-PHAGE is presented in Figure 1. Some parameters such as directory locations, database connection parameters, and program options are system specific hence they require customization. These parameters are defined in the Config directory and are not hard-coded in the software implementation. The scripts refer to this directory for obtaining the parameters required for their execution. Understanding and implementing these steps will require some technical expertise with the UNIX/Linux operating systems. A user manual is also provided that explains the use of various features.

SNP-PHAGE software was implemented with the following features.

**Figure 2:**
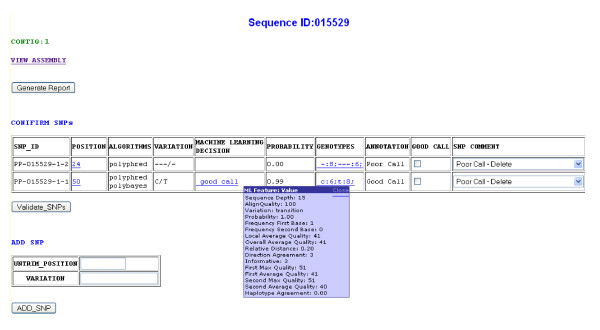
**Screenshot of SNP-PHAGE graphical interface**. For making SNP validation decisions this interface provides links for viewing global and local sequence alignment, genotypes along with phred quality scores, machine learning (ML) inference and ML feature values and checkbox/pop down menu to mark individual SNP as being a good/poor call.

• A standard chromatogram naming convention is required to facilitate sequence assembly by following the St. Louis convention described in the Phrap documentation [2] for easy association with sequence tag sites and individual genotypes. A file renaming script (renameFiles.pl) is also provided with this software for help with filename conversion.

• Phred was used for base calling because it provides quality scores along with the base calls. While our internal test runs (not reported) with the demo version of the TraceTuner commercial software provided similar results as Phred for high quality bases, the two base-calling software packages gave variable results with low quality bases. Neither software was consistently superior.

• Phrap was used for sequence assembly of chromatograms from STSs, because it has more flexible parameters than CAP3 [21] to force alignment at lower match scores.

• The SNP discovery pipeline includes: 1) PolyBayes, which uses a Bayesian inference engine to calculate the probability that a given site is polymorphic primarily optimized for detecting SNPs among homozygous individuals; and 2) PolyPhred, which is optimized for detecting SNPs in heterozygous individuals. PolyBayes software has been used to accurately predict SNPs in humans with EST sequences aligned to finished and working-draft quality genomic sequences[22]. It can also be used to analyze amplified STSs from inbred species such as soybean [23].

• Chromatograms from several STSs can be combined into a batch for automated analysis. The output files from PolyBayes and PolyPhred containing the candidate polymorphisms are parsed and entries are made to a relational database.

• A web interface provides access to search or browse options through a list of STSs to analyze and validate candidate polymorphisms (Figure 2). Additional options include the ability to edit the consensus sequence, add new SNPs, and to generate reports for each STS containing confirmed SNPs.

• SNP validation can be performed for local or global alignments with candidate SNPs visualized from a web based graphical interface similar to the Consed [24] output screen. The quality of the base at the polymorphism position can be ascertained from the background color of the base on the screen as in Consed.

• The SNP-PHAGE software currently has a limitation in viewing the underlying trace files. Hence, heterozygous SNPs marked by PolyPhred have to be visualized using the Consed interface. A software update to incorporate this feature will be released at a later date.

• The results generated from a machine learning algorithm along with the multiple features used in the analysis are displayed to aid the decision making process.

• Candidate polymorphisms can be either confirmed or eliminated from the web interface after manual inspection. These expert-confirmed polymorphisms are then used to create new database entries of validated SNPs, to assign a genotype for each sample, to identify haplotypes within an STS, and to make database entries to facilitate dbSNP submissions.

## Results and discussion

SNP-PHAGE has been applied to amplified STSs of soybean to discover SNPs [23]. Following studies have used SNP-PHAGE for the discovery of over 10,000 SNPs in soybean STSs. A subset of 1,185 SNPs from this set was used to generate a high density soybean linkage map (manuscript in preparation). SNP-PHAGE was also used in comparative legume analysis. A set of 1,165 soybean primers were used to amplify genomic regions from seven legumes and resulting inter-species polymorphisms were used to study the evolutionary relationships between these legumes (manuscript in preparation). SNP-PHAGE was also used to validate *in-silico *predicted SNPs in soybean using EST data (manuscript in preparation).

SNP-PHAGE is designed to identify a set of putative SNPs by applying PolyBayes and PolyPhred. PolyBayes and PolyPhred were optimized for different SNP detection scenarios (homozygous and heterozygous SNPs) and inclusion of both packages in the software pipeline enhances flexibility. Depending on the species or DNA/RNA source considered, one of the algorithms may be more appropriate. Application of machine learning in polymorphism discovery was shown to improve the efficiency of SNP discovery by 85% with SNPs predicted by PolyBayes. Inclusion of machine learning inference along with a visual interface for easy and quick visualization of SNPs is likely to also improve the efficiency of SNP validation.

SNP-PHAGE accounts for differences in SNPs detected by PolyBayes and PolyPhred. When a SNP is called by both algorithms, genotypes generated from PolyPhred are used, because it finds both homozygous and heterozygous bases. PolyBayes marks each position in a multiple indel as a separate SNP, whereas PolyPhred combines the multiple indels into a single SNP, as is required for dbSNP submission. The SNP positions in such multiple indels from PolyBayes can be flagged during SNP validation so that they can be combined into a single indel in subsequent analysis. The consensus sequence at SNP positions depends on the individuals genotyped, because bases in those positions are derived from the most frequently observed genotypes. For indels, the change is more striking to the user, because it can offset other SNP positions in the consensus sequence.

SNP-PHAGE can, in principle, be extended to work with other UNIX/Linux based SNP detection packages such as novoSNP [11] and SNPdetector [12]. However, these packages use different sequence matching and assembly algorithms, including programs such as BLAST and SIM that may result in different alignments, and thus SNP positions may differ.

The interface allows the user to deal with some exceptional cases encountered during the expert SNP validation such as (i) identification of a high quality SNP not called by the prediction programs, (2) poor consensus sequence trimming, and (3) an incorrect consensus base at the SNP position. The interface allows for the addition of the SNP, along with the entry of the associated genotype into the database, custom consensus sequence trimming, and editing of consensus sequence bases.

## Conclusion

SNP-PHAGE is a simple, user friendly package for automated high-throughput polymorphism discovery. The package is provided with an installation guide and user manual to assist in deployment and implementation of this application. Additional pre-requisites for installing this application are minimal and only require those needed for base calling, sequence assembly and polymorphism detection.

Polymorphism discovery and validation requires a balance between sensitivity (minimize the false negative SNPs) and specificity (minimize the false positive SNPs). However, these requirements may vary for individual projects. Projects driven by the need to reduce sequencing costs may wish to investigate all putative SNPs thus allowing for false positives; whereas other targeted projects such as those using SNPs as a biomarker for disease validation cannot tolerate false positives. The polymorphism detection programs attempt to fulfill these requirements by ranking SNPs by a score/probability value. The number of false positive SNPs predicted by PolyBayes and PolyPhred has been shown to be reduced by additionally considering the alignment and quality of the neighboring bases (NQS [4]). The modified version of NQS has been used in SNPDetector [12]; whereas, in the machine learning (ML) approach, the required feature values are automatically determined from an expert-annotated dataset [17]. SNP-PHAGE software calculates and makes database entries for a number of additional features not implemented by other SNP detection software. These features can also be used to generate a new custom set of rules for filtering SNPs. ML was applied only on the SNPs detected by PolyBayes. One of the features that can be added for heterozygous SNPs is the ratio of peak heights, as SNPdetector implementation requires this ratio to be at least 0.3.

SNP-PHAGE provides the basic framework for SNP detection and validation. Other supplementary features and functions are being considered for addition to the interface. These additional features will be provided as updates in future with notifications by email for all registered users. This open source resource is intended to be further improved through user suggestions and encourages participation through provision of their custom scripts to be included in SNP-PHAGE. Some of these features planned will require availability of additional data such as whole genome sequence to provide genome coordinates, EST sequence to annotate the location of intron and exon sites, and protein sequence for annotation as synonymous vs. non-synonymous SNPs. Other scripts that are being customized for incorporation into the SNP-PHAGE include trio validation, estimation of θ and Fst values [25] and data formatting to provide data in the formats required for performing other analyses such as Phylogeny, and PHASE [26] or HaploView [27].

## Availability and requirements

Project name: SNP-PHAGE

Software: Additional file 1

(Check the project home page for more recent versions)

Test dataset: Additional file 2

Project home page: 

Operating system: UNIX/Linux

Programming language: Perl

Other requirements: Phred, Phrap, Consed, MySQL and Perl modules from CPAN listed in the project documentation (installation guide).

License: Open GPL

Any restrictions to use by non-academics: None

## Authors' contributions

LM, JG dealt with the computational aspects in the implementation

IC, DH and PC performed the sequencing, SNP data generation and suggested important features.

CVT provided overall guidance for this project

All authors read and approved the final manuscript.

## Supplementary Material

Additional file 1 SNP-PHAGE software package. This compressed file contains all scripts required to create a SNP processing pipeline and a web interface for data analysis and visualization that is powered by a backend relational database. Click here for file

Additional file 2 Test dataset. This compressed file contains test data for recreating the demo website. It contains the database table entries and chromatogram files to test for proper installation of all functionalities provided. Click here for file
