# Supplementary material for: SNP-PHAGE – High throughput SNP discovery pipeline
Source: BMC Bioinformatics. 2006 Oct 23;7:468. doi: 10.1186/1471-2105-7-468 (PMC1626092; doi:10.1186/1471-2105-7-468)
Supplement: Additional file 1 — SNP-PHAGE software package. This compressed file contains all scripts required to create a SNP processing pipeline and a web interface for data analysis and visualization that is powered by a backend relational database. [file 1471-2105-7-468-S1.gz › Software/HTML/footer.html]

For questions regarding the ***SNP-PHAGE*** 
software please send a email to

Lakshmi K Matukumalli
