# Supplementary material for: SNP-PHAGE – High throughput SNP discovery pipeline
Source: BMC Bioinformatics. 2006 Oct 23;7:468. doi: 10.1186/1471-2105-7-468 (PMC1626092; doi:10.1186/1471-2105-7-468)
Supplement: Additional file 1 — SNP-PHAGE software package. This compressed file contains all scripts required to create a SNP processing pipeline and a web interface for data analysis and visualization that is powered by a backend relational database. [file 1471-2105-7-468-S1.gz › Software/HTML/header.html]

SNP-PHAGE 


|  |
| --- |
| SNP-PHAGE |
|  |

|  |  |
| --- | --- |
|  |  |
| Introduction  Home  SNP  SNP Detection  Machine Learning  ML-Implementation  ML-Optimization  Links  SNP-PHAGE  Flow Chart  User Manual  Database Tables  Browse SNP Database  dbSNP submissions  Applications |  |
