# Supplementary material for: SNP-PHAGE – High throughput SNP discovery pipeline
Source: BMC Bioinformatics. 2006 Oct 23;7:468. doi: 10.1186/1471-2105-7-468 (PMC1626092; doi:10.1186/1471-2105-7-468)
Supplement: Additional file 1 — SNP-PHAGE software package. This compressed file contains all scripts required to create a SNP processing pipeline and a web interface for data analysis and visualization that is powered by a backend relational database. [file 1471-2105-7-468-S1.gz › Software/HTML/UserManual.pdf]

SNP-PHAGE Version 1.1

User Manual

Lakshmi K Matukumalli
March 2006

Contents

1. Introduction.......... 3
2. Renaming chromatogram files.......... 4
3. Chromatogram files processing.......... 5
4. Data analysis from Web Interface.......... 6

Figures

Figure 1: Flow Chart of SNP-PHAGE.......... 3
Figure 2: SNPs Summary of all STS.......... 7
Figure 3: View Putative SNPs.......... 8
Figure 4: Confirm SNPs.......... 9
Figure 5: Application of Machine Learning.......... 10
Figure 6: View Sample Genotypes.......... 11
Figure 7: Global Sequence Assembly.......... 12
Figure 8: Local Sequence Assembly.......... 13
Figure 9: Add SNPs.......... 14
Figure 10: Edit Consensus Sequence.......... 15
Figure 11: Sequence Primers and Annotation.......... 16
Figure 12: Confirmed SNPs, Consensus Sequence and Haplotypes.......... 17

1. Introduction

SNP-PHAGE software attempts to simplify and streamline data processing and analysis tasks and to provide a web-interface with a backend database for investigators to perform intelligent queries about their data. SNP-PHAGE software is a data analysis pipeline that executes several programs (open source / free for academic use) in sequence for SNP detection. This package also includes a machine learning component developed by this group to assist in making decisions about putative SNPs.

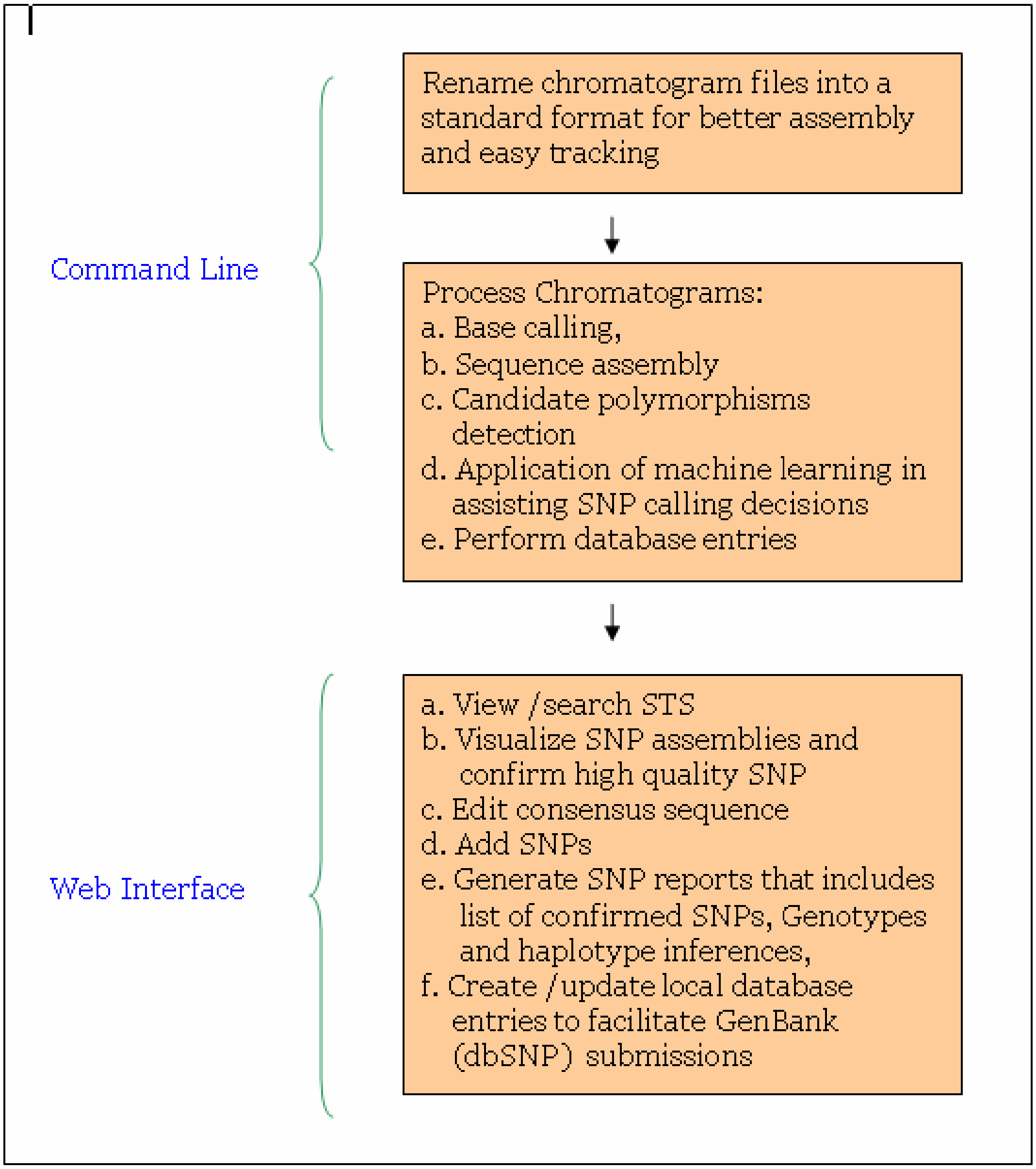

Figure 1: Flow Chart of SNP-PHAGE
Polymorphisms analysis of multiple sequence tag sites using SNP-PHAGE is effectively a three stage process where the first two steps are performed from command line interface with the final step from a user-friendly web interface.

2. Renaming chromatogram files

Analyzing data through this software pipeline requires chromatogram files renaming into a format that is recognized by the sequence assemblers for making better contigs and also for efficient data management through this interface. A file renaming script along with a working example is provided to help users through the process. Each sequence tag site (STS) is encoded by a 6 character long alpha-numeric string to provide a sufficiently large number of tags. Use of lower and upper case characters to distinguish STS should be avoided as some of the queries performed to enable case-insensitive searches. Each genotyped sample (individual) is identified by a 7 character long alpha-numeric string. These characters can be further split by using the first 3 characters to identify a population and the last 4 characters for identifying the individual sample within the population.

The file name has three necessary parts

The filename has three necessary parts that are necessary for identification

(i) STS identifier (6 alpha numeric characters)
(ii) Individual name (7 alpha numeric characters)
(iii) Direction of the read (b or g depending on the direction of the read)

For example for the chromatogram
P13_035397JaloEEP.b_01.ab1

035397 is the STS identification number
JaloEEP is the identifier name and
b represents the direction of the read.

The values P13 and 01 are the details about to the well location and lane number of the sequencing run and are not used by the program further.

P13 represents the well location on the sequencing plate (optional) and
01 is the lane number of the sequencer capillary (optional).

The package includes a Perl script ***renameFiles.pl*** that takes two arguments

1. A tab delimited file containing 4 fields for each column that are (current file name and STS identifier, individual name and direction of the read)
2. Option to copy /move files into a new folder after renaming.
3. Option to save old/new file names to a file

Program usage : renameFiles.pl <FILE_NAME> <copy/move> <save Old/New FileNames yes/no>

<file_name>

The file should contain the following information in the tab delimited format

1. Current File name (along with the path)
2. STS identifier (upto 6 characters in length --rest will be trimmed)
3. Individual Identifier for eg., Breed, Cultivar,Population or Person Name
(upto 7 characters in length --rest will be trimmed)
4. Read Direction (Forward / Reverse)

<copy/move>

copy -- To rename the file and new file is stored in another directory
move -- To move the original file into another directory after renaming

<save Old/New FileNames>

yes -- saves the new and old file names to a file
no -- show the new and old files only on the screen

3 sample files are included in the testdata/ ChromatsDirBeforeRenaming that can be modified by running this script as

perl renameFiles.pl TestRename.txt copy yes

with names not following the convention. A file TestRename.txt containing the old file name and the

3. Chromatogram files processing

Multiple STS can be processed in a single batch. The perl script batch_process.pl takes input file containing sequence IDs as argument. For example the test_data containg 6 STS can be processed by running the following command

$ perl batch_process.pl IDFILE.txt

The output will be as follows:

run_phredPhrap.pl 015471

POLYPHRED Version 5.04

Reading the ACE file
/data2/lmatukum/Software/SNP_PHAGE/test_data/TempDataStore/edit_dir/015471.fasta.screen.ace.1
Reading the PHD and POLY files.
  Reading information for contig Contig1
Processing the contig Contig1
Searching for SNPs
Searching for insertion / deletion sites.

Updating the ACE file
/data2/lmatukum/Software/SNP_PHAGE/test_data/TempDataStore/edit_dir/015471.fasta
.screen.ace.1
Updating the PHD files.
  Updating information for Contig1
Writing to standard output.

Finished 015471Renaming Files
The batch process script executes several processes in sequence such as base calling (phred), sequence assembly (phrap), polymorphism detection (polyphred and polybayes) and application of machine learning algorithm on all putative SNPs. Also the results from all these processes is parsed and populated into a MySQL relational database to enable user to perform intelligent queries about their data.

4. Data analysis from Web Interface

The web output for data analysis tasks is mostly straight forward. However explanations are provided to guide the users through the different features within each screen to help better utilize this resource. The web interface and the queries included only provide the basic frame work for data analysis. More development towards the database and web screens will be needed to answer specific questions relevant for the individual groups.

Some such possible data analysis extensions analysis with additional data inputs from other sources (if available) are

1. SNP query by genome locations.
2. Identification of Introns / Exons in a sequence tag site
3. Marking synonymous / non-synonymous SNPs
4. Estimation of θ
5. Fst / LD analysis

STS Entries with SNP Counts

SEQID	polybayes	manual	polyphred	Confirmed	View Assembly	View Putative SNPs	Edit SNPs	Generate Report
015529	1	1	2	1	Contig1	VIEW	EDIT	REPORT
015501	91		12	4	Contig1	VIEW	EDIT	REPORT
015471	19		8	8	Contig1	VIEW	EDIT	REPORT
015903	1			1	Contig1	VIEW	EDIT	REPORT
015493	5		1	1	Contig1	VIEW	EDIT	REPORT

Show 20 Entries per Page
Pages: 1

Figure 2: SNPs Summary of all STS

The screenshot above displays the summary of putative SNPs identified and confirmed for each STS. This screen has the following main features numbered above

1. Count of SNPs identified by the SNP detection programs PolyBayes and PolyPhred and the number of SNPs that have been manually added.
2. Count of SNPs confirmed by an expert
3. Link to view global sequence assembly of each contig (Figure 7)
4. Link to view all putative SNPs (Figure 3)
5. Link to confirm SNPs (Figure 3) and edit consensus sequence (Figure 10)
6. Link to view report after SNP validation (Figure 12)

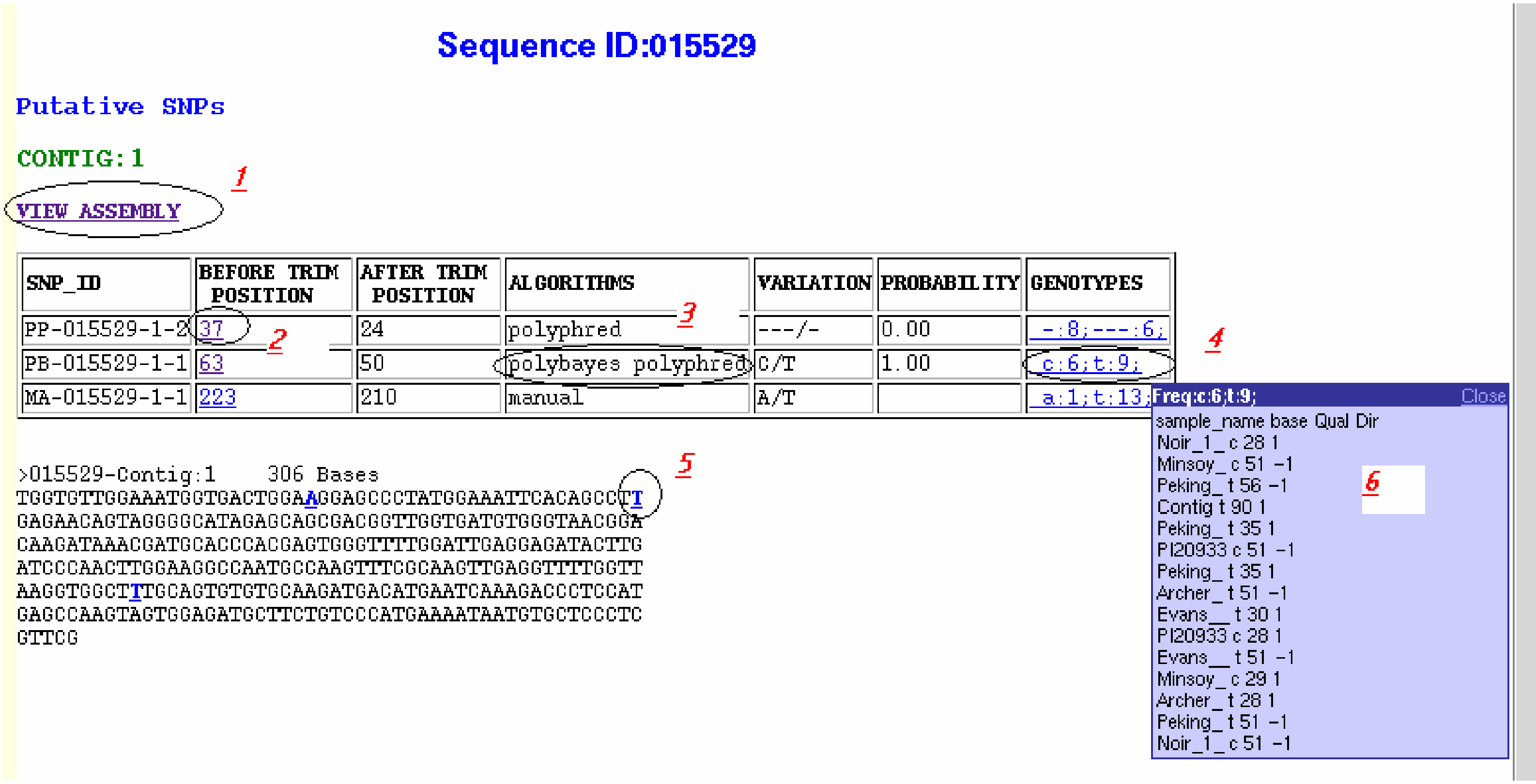

Figure 3: View Putative SNPs

This screenshot shows a list of putative SNP for each STS. The first two characters of the SNP ID codes for prediction algorithm (PB –PolyBayes, PP-PolyPhred and MA-Manual). Subsequent characters code for the STS ID, contig number and a serial number (incremented for each SNP predicted by individual algorithms). This screen has the following important features numbered above.

1, 2 : Links to view global (Figure 7) and local sequence assemblies (Figure 8)

3: Algorithm that predicted the SNP (multiple predictions for the same position are clustered)

4, 6: View genotypes for each sample at a given SNP locus along with phred score.

5: SNP location in the consensus sequence.

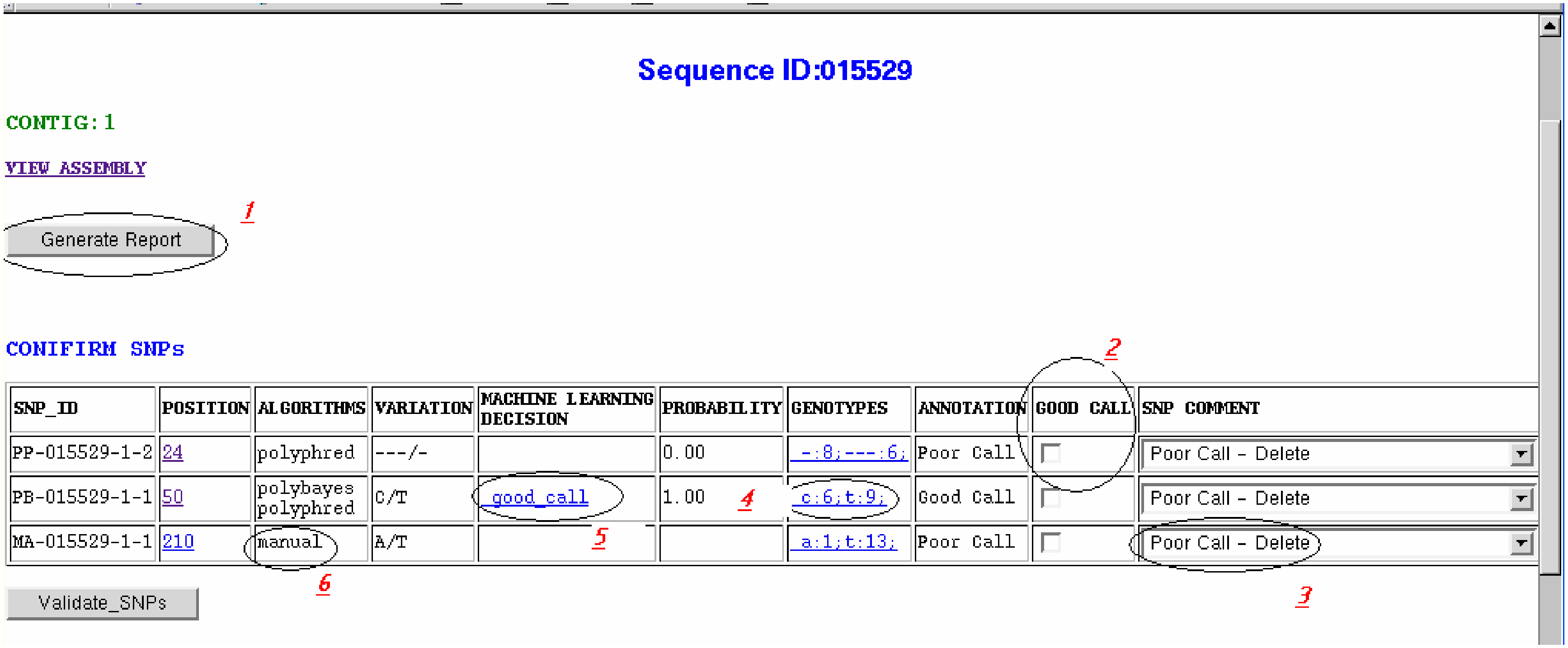

Figure 4: Confirm SNPs

This screen shot shows the interface to annotate a SNP as a good call or a poor call. The important features of this screen are numbered above

2,3 : A SNP can be confirmed either by marking the checkbox in 2 or by selecting the appropriate comment. A SNP that is un-marked with a comment of Poor Call –Delete will be considered a poor call.

4, 5: To aid the validation the individual genotypes can be visualized (Figure 5) and the ML features (Figure 5) along with the ML algorithm result are shown.

6: SNPs manually added are also visible from this interface with an option to accept or ignore the SNP.

1: After performing the SNP validation a SNP report can be generated.

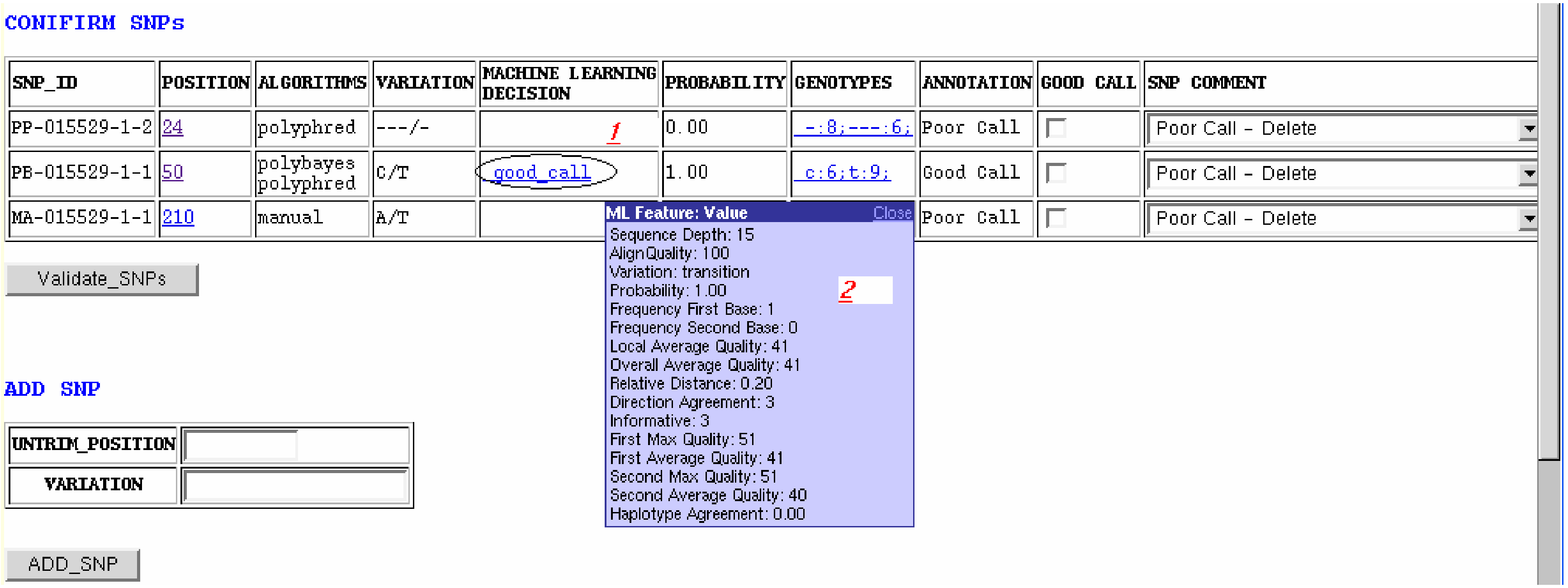

Figure 5: Application of Machine Learning

To aid the expert in SNP validation machine learning (ML) algorithm was applied for the SNPs detected by PolyBayes. The screen shot shows the result (1) from application of ML. The features and the values used for applying ML to identify high quality SNPs is also shown (2)

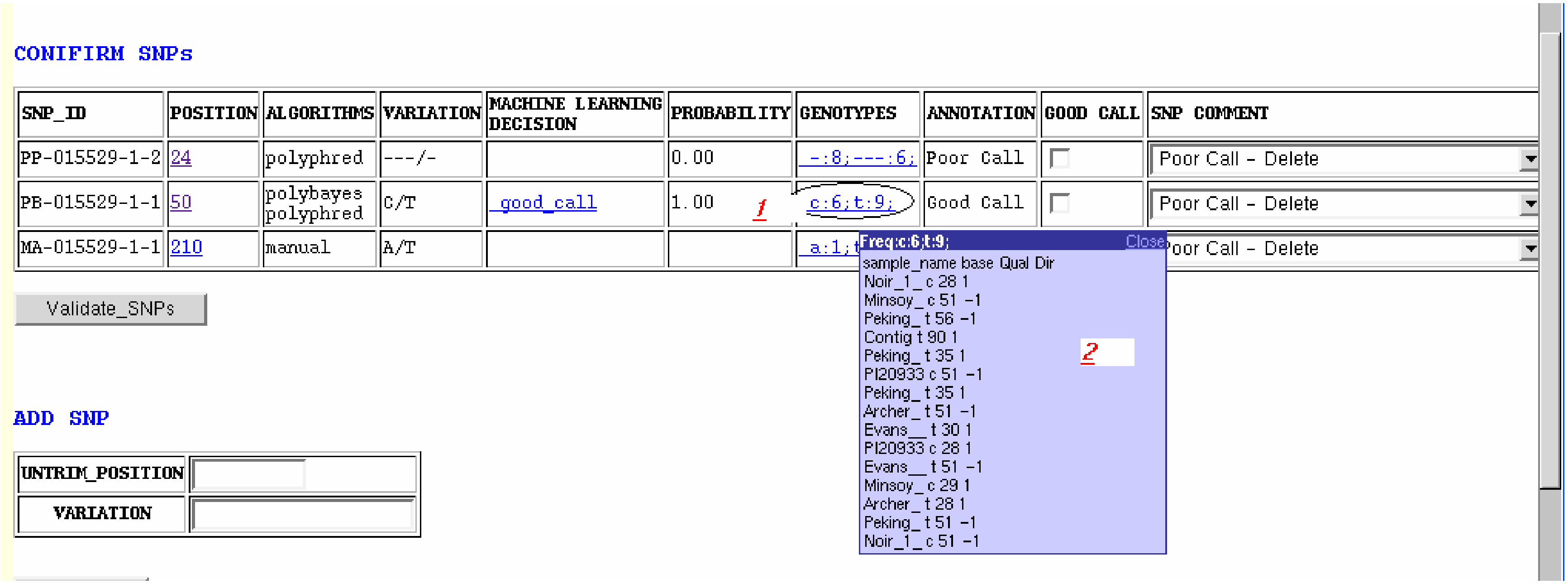

Figure 6: View Sample Genotypes

To make a decision about a SNP the frequency of occurrence of variable base call, the quality of the base calls and agreement of the base call in chromatogram sequenced from both directions for a given sample is important. The frequency of occurrence (1) and individual base call information from different reads is shown in the pop down window (2).

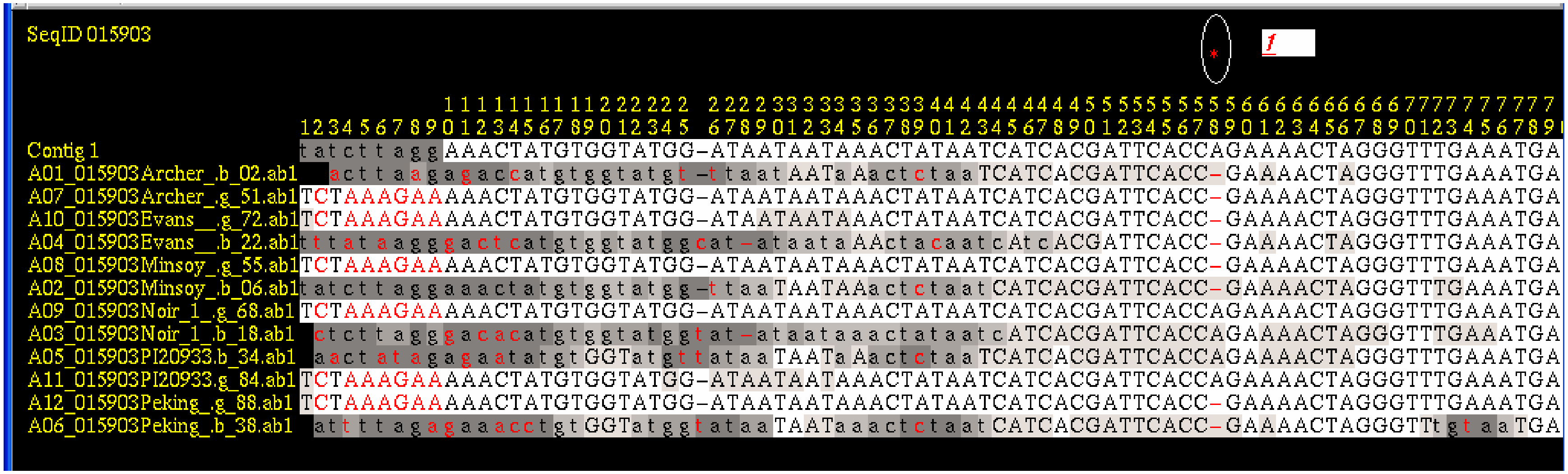

Figure 7: Global Sequence Assembly

The screenshot shows the global sequence alignment of a STS. The visualization is similar to that of a Consed. The background color of the base is indicative of the phred score. While the uppercase bases with white background and represent the highest quality and the bases in lower case with dark background represent very low sequence quality. The consensus sequence and base positions are indicated on the top. The SNP positions are marked with a star above the alignments (1). The reads are clustered by the sample name such that reads in both directions appear together to help assess their directional agreement at the SNP position. However, this web output does not have the ability to show the underlying sequence trace as in Consed.

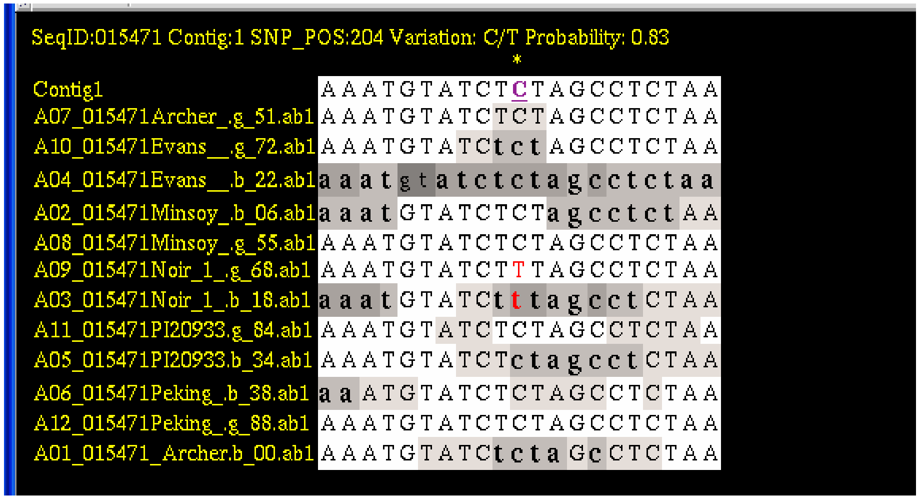

Figure 8: Local Sequence Assembly

The screenshot shows the local sequence alignment of a STS around a SNP of interest. The visualization is similar to that of a Consed. The background color of the base is indicative of the phred score. While the uppercase bases with white background and represent the highest quality and the bases in lower case with dark background represent very low sequence quality. The consensus sequence and base positions are indicated on the top. The SNP positions are marked with a star above the alignments (1). The reads are clustered by the sample name such that reads in both directions appear together to help assess their directional agreement at the SNP position. However, this web output does not have the ability to show the underlying sequence trace as in Consed.

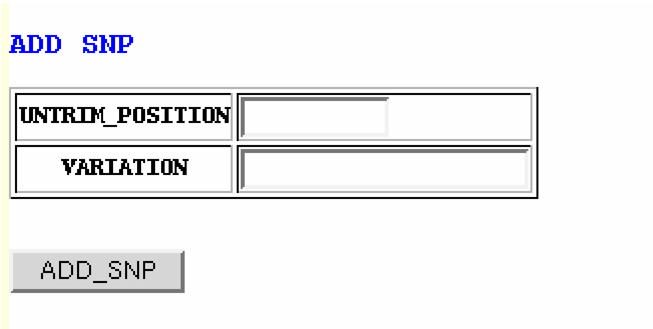

Figure 9: Add SNPs

All variant positions are analyzed by the detection programs and filtered by different criteria to assess for high quality SNPs. However, an investigator may want to call a variant position to be called a valid SNP that is not identified by the software. This functionality will be useful in such circumstances. Adding a SNP from this interface will also add the associated sample genotypes in the genotypes table.

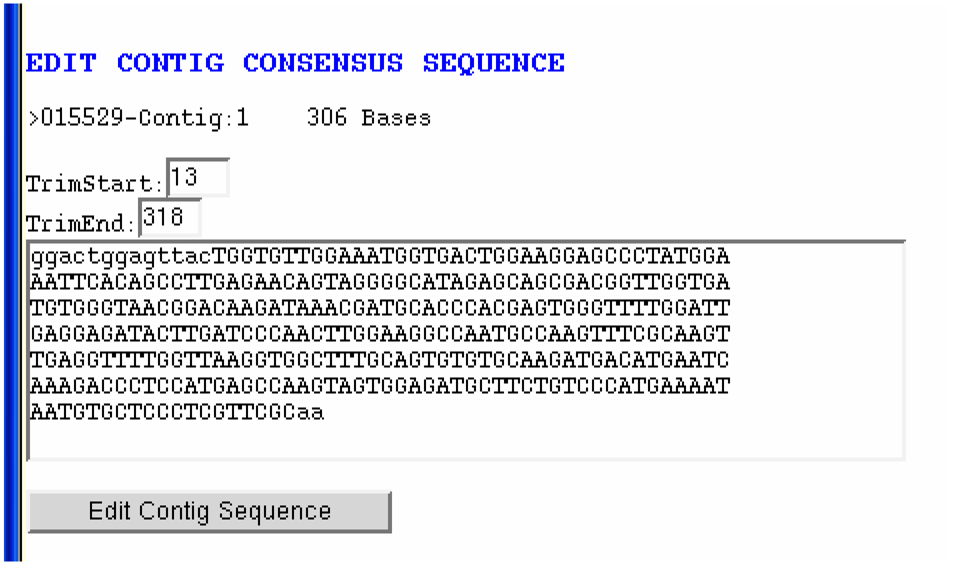

Figure 10: Edit Consensus Sequence

This feature will be useful for choosing alternate positions for sequence trimming and edit the consensus base. Sequence trimming by the programs is usually performed by finding at least one base with sequence quality above a given value. However, in some cases there may be long stretch of poor quality bases after one or two high quality bases that will require alternate trim co-ordinates. For the consensus sequence the sequence assembly program chooses one or the other base at the SNP positions. To select a consensus base at the SNP positions this feature will be useful.

Sequence ID:015529

Generate Report

PERSON	Genbank Accession	Forward Primer	Reverse Primer	Annotation
Ik–Young	AW349710	GCGAACGAGGGAGCACATTATTTT	GCGAAGGTGGATGTTTATAGTTATG	similar to PIR–S10930–S10930 S–receptor kinase homolog PK1 precursor – maize {Zea mays;} , partial (9%)

Figure 11: Sequence Primers and Annotation

This is part of the report that shows the forward and reverse primers, the source sequence used to develop these primers and annotation for the sequence tag site. This screen can be customized to show the chromosome locations for the sequenced genomes.

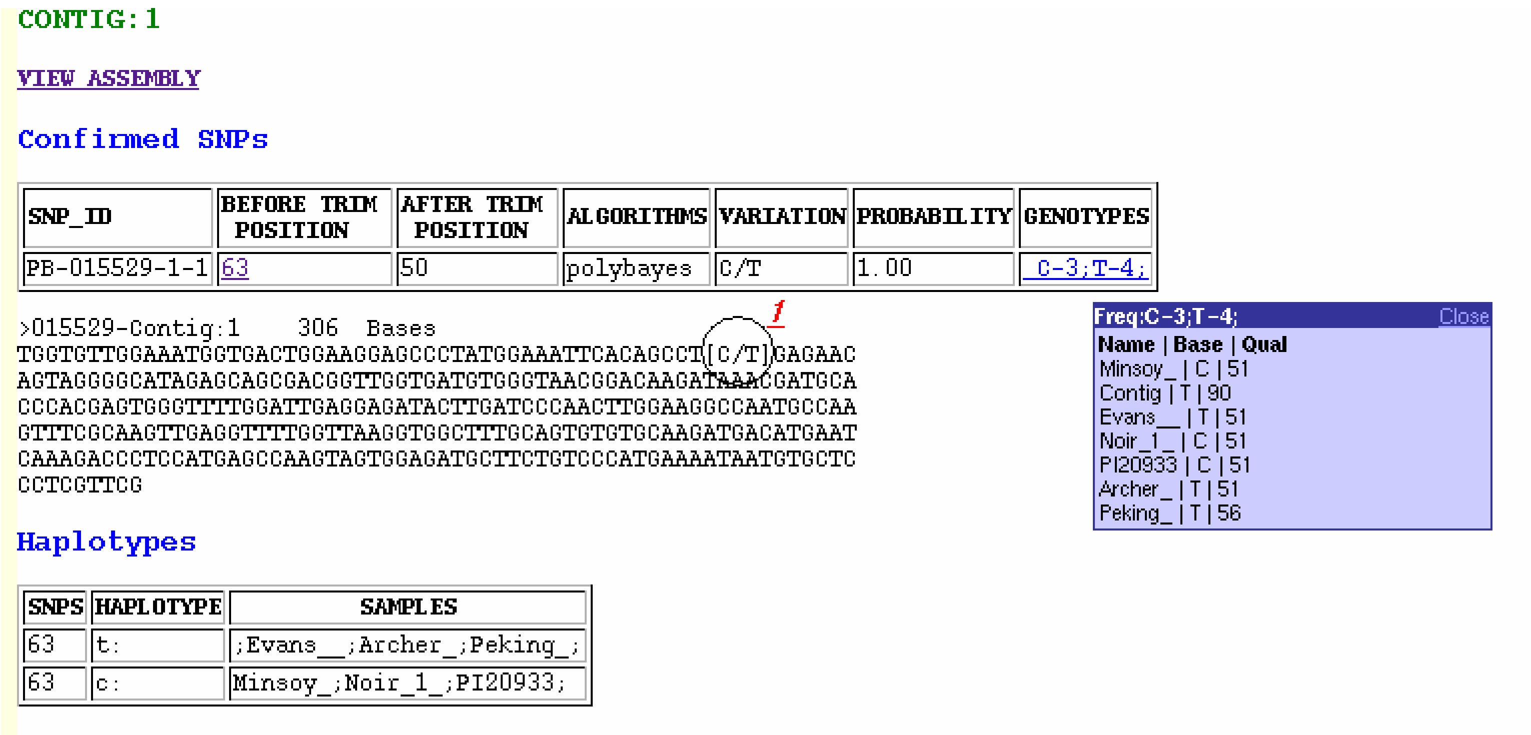
CONTIG:1

VIEW ASSEMBLY

Confirmed SNPs

SNP_ID	BEFORE TRIM POSITION	AFTER TRIM POSITION	ALGORITHMS	VARIATION	PROBABILITY	GENOTYPES
PB-015529-1-1	63	50	polybayes	C/T	1.00	C-3;T-4;

```
>015529-Contig:1    306  Bases
TGGTGTTGGAAATGGTGACTGGAAGGAGCCCTATGGAAATTCACAGCCT[C/T]GAGAAC
AGTAGGGGCATAGAGCAGCGACGGTTGGTGATGTGGGTAACGGACAAGATAAACGATGCA
CCCACGAGTGGGTTTTGGATTGAGGAGATACTTGATCCCAACTTGGAAGGCCAATGCCAA
GTTTCGCAAGTTGAGGTTTTGGTTAAGGTGGCTTTGCAGTGTGTGCAAGATGACATGAAT
CAAAGACCCTCCATGAGCCAAGTAGTGGAGATGCTTCTGTCCCATGAAAATAATGTGCTC
CCTCGTTCG
```

1

Freq:C-3;T-4; Close

Name	Base	Qual
Minsoy_	C	51
Contig	T	90
Evans__	T	51
Noir_1_	C	51
PI20933	C	51
Archer_	T	51
Peking_	T	56

Haplotypes

SNPS	HAPLOTYPE	SAMPLES
63	t:	;Evans__;Archer_;Peking_;
63	c:	Minsoy_;Noir_1_;PI20933;

Figure 12: Confirmed SNPs, Consensus Sequence and Haplotypes

The fields in this screen will be generated / refreshed when the GenerateReport button is pressed. The existing entries in the database are removed and new entries are made. The entries include new consensus sequence with the polymorphism (1). One genotype per sample is shown that is from the highest quality read available at the SNP position. The haplotype report shows all the haplotypes generated from the confirmed SNPs and cluster samples by the haplotype.
