# Supplementary material for: SNP-PHAGE – High throughput SNP discovery pipeline
Source: BMC Bioinformatics. 2006 Oct 23;7:468. doi: 10.1186/1471-2105-7-468 (PMC1626092; doi:10.1186/1471-2105-7-468)
Supplement: Additional file 1 — SNP-PHAGE software package. This compressed file contains all scripts required to create a SNP processing pipeline and a web interface for data analysis and visualization that is powered by a backend relational database. [file 1471-2105-7-468-S1.gz › Software/HTML/DatabaseTables.pdf]

USERS
USER
PASSWORD

CONTIGS
**SEQID**
CONTIG_No
CONTIG_LEN
TRIM_LEFT_POS
TRIM_RIGHT_POS
CONTIG_SEQ
EDITED
QUALITY
SEQUENCE

PRIMERS
NAME
**SEQID**
SOURCE_DNA_SEQ
SOURCE_PROTEIN_SEQ
GB_ACC
FP
RP

CHROMATS
SAMPLE_CHROMAT
**SEQID**
SAMPLE_NAME
READ_DIRECTION
TRIM_START
TRIM_END
SEQUENCE
QUALITY
SEQ_LENGTH
CONTIG_No

SAMPLE_GENOTYPES
**SEQID**
SNP_ID
SAMPLE_CHROMAT
SAMPLE_NAME
SNP_POSITION_CHROMAT
BASE
BASE_QUALITY
SNP_QUALITY
DIRECTION

PUTATIVE_SNPS
**SEQID**
**SNP_ID**
CONTIG_NO
CONTIG_LENGTH
UNPAD_POSITION
PAD_POSITION
VARIATION_TYPE
TEMPLATE_DEPTH
SEQUENCE_DEPTH
PROBABILITY
ALGORITHM
DATE

ML_Features
**SNP_ID**
Seq_Depth
Align_Quality
var_type
Probability
Freq_base_first
Freq_base_second
Local_Avg_Qual
Overall_Avg_Qual
Rel_distance
Dir_Agreement
Informative
First_max_qual
First_avg_qual
Second_max_qual
Second_avg_qual
Haplotype
ML_Decision
Expert_Decision

CONFIRMED_SNPS
**SEQID**
**SNP_ID**
SNP_POS_BF_TRIM
SNP_POS_AF_TRIM
SNP_PROB
SNP_TYPE
SEQ_5_PRIME
SEQ_3_PRIME
CONSENSUS_SEQ
MODIFICATION
CONSENSUS_LENGTH
USER
SNP_COMMENT

SAMPLE_HIGH CONFIDENCE_GENOTYPES
**SEQID**
SNP_ID
SAMPLE_NAME
BASE
BASE_QUALITY

Haplotypes
**SEQID**
CONTIG_NO
SNPs
Haplotype
Samples

SNP_GENBANK_DETAILS
SNP_NUMBER
**SEQID**
**SNP_ID**
GB_SUBMISSION_DATE
DB_ENTRY_DATE
GB_SNP_ID
SUBMITTER
GB_ACC
PRODUCT_TYPE
SEQUENCE_SOURCE
PHENOTYPE_ASSOCIATION
COMMENT
PRIMARY
