# Supplementary material for: SNP-PHAGE – High throughput SNP discovery pipeline
Source: BMC Bioinformatics. 2006 Oct 23;7:468. doi: 10.1186/1471-2105-7-468 (PMC1626092; doi:10.1186/1471-2105-7-468)
Supplement: Additional file 1 — SNP-PHAGE software package. This compressed file contains all scripts required to create a SNP processing pipeline and a web interface for data analysis and visualization that is powered by a backend relational database. [file 1471-2105-7-468-S1.gz › Software/InstallationGuide.pdf]

SNP-PHAGE Version 1.1

Installation Guide

Lakshmi K Matukumalli
October 2006

Contents

1. Introduction 3
2. Pre-requisites Software and Hardware (Dependencies) 3
3. Custom configuration 4
    3.1 Create database tables 4
    3.2. Database connections. 5
    3.3. Directory locations 5
4. Checking installation 6
5. Checking installation 5. Setting up Web Interface 7
5. Setting up Web Interface 7
6. Renaming chromatogram files 8
7. Processing Files 9

1. Introduction

SNP-PHAGE package helps in setting up a SNP discovery pipeline with database and user friendly web-interface functionalities for editing and querying the polymorphism data. SNP-PHAGE is designed for use with most widely used (*de-facto standard*) bioinformatics programs for SNP discovery ie., PolyBayes and PolyPhred with planned extensions to add SNPDiscover software into the pipeline in the recent future.

SNP-PHAGE software is designed to be used on Unix/Linux platforms principally limited by the other software dependencies that can only run on these systems. SNP-PHAGE is primarily designed for use with MySQL database and some additional effort may be required for users to port on other databases. CGI-perl is used to generate dynamic HTML pages.

Test scripts are provided to check for the installation and provide familiarity to help custom configure for specific needs.

2. Pre-requisites Software and Hardware (Dependencies)

Operating System: Due to dependency on Phred this software can only be implemented in Linux/UNIX like systems. This cannot be implemented in windows. Please check this site (http://www.phrap.org/consed/consed.html#howToGet) if Phred is available for the specific OS you are looking for.

Operating System Version: This software was developed on Redhat Linux. The versions tested are 7.3 through 9. However, other UNIX/Linux based OS will generally be able to implement this software. Please check the installation support for the Bioperl package for your specific operating system version. (http://www.bioperl.org/wiki/Getting_BioPerl)

MySQL database Version: MySQL version 4.0 or above are compatible with this software.

Space Requirements: The space requirements for SNP-PHAGE package scripts are modest (less than 1 MB). However for processing and storage of chromatograms you would require hard disk space of several GB depending on the scale of your project.

Memory Requirements: The memory requirements of SNP-PHAGE scripts itself are very low. However you require higher memory for sequence alignments using Phrap that depends on the number of reads per run. Please check the phrap documentation (http://www.phrap.org/phredphrap/phrap.html) for details.

The following free for academic use bioinformatics software is required for this analysis. The website links where they can be obtained are given below.

1. **perl 5.8 and above** *(http://www.perl.org)*
2. **Phred/Phrap/Consed** *(http://www.phrap.org/)*
3. **PolyBayes** *(http://genomeold.wustl.edu/groups/informatics/software/polybayes/)*
4. **PolyPhred** *(http://droog.gs.washington.edu/PolyPhred.html)*
5. **C4.5** *(http://www.rulequest.com/Personal/c4.5r8.tar.gz)*

Installation of free open-source MySQL database: (http://www.mysql.com)

Perl modules from CPAN (http://www.cpan.org)

1. **Bundle::Bioperl**
2. **Bundle::Expect**
3. **Expect**
4. **DBI**
5. **Class::MethodMaker**
6. **CGI**
7. **Statistics::Descriptive**

CPAN modules can be easily installed by querying and installing from the command line by using the command

perl -MCPAN –e shell

After logon to cpan shell search for the latest version using the command

cpan> i /Bundle-Bioperl/

The search will result in the latest version. You can then select the package and install.

Guidelines for installing the software and support is generally offered by the individual providers, however we can provide guidance and share our experience for any specific problems users may be having in installing the above software.

3. Custom configuration

3.1 Create database tables

The mysql database schema is provided in the file Software/CreateTables.sql. You have to login to create to create two new blank databases one for test and other for your production server. The names for these databases are optional. The tables in the new production database can be created by running the following command from the command prompt.

mysql –u user_name –p production_database_name < CreateTables.sql

Similarly a test database can be created by running the following command from the command line using the script provided test_data/snp_phage_test.sql. This file contains both database tables and data for some amplicons processed through this pipeline before.

mysql –u user_name –p test_database_name < snp_phage_test.sql

The test database will be useful to checking the functionality of the web interface scripts for the first time and also for experimentation with new changes and future updates.

3.2. Database connections.

Database parameters for making the connections have to be specified in two different files (i) for the web interface to display data and (ii) for the command line to make entries while data processing.

For the command line interface you have to specify in the file Software/Config/myDatabase.pm and for the web interface you have to specify in the Software/cgi-bin/myDatabase.pm directory. The following parameters have to be provided for making the data connections. (Note these files will have to be copied to other destinations see below)

-- Database Name
-- Username
-- Password

3.3. Directory locations

The SNP-PHAGE software itself can be run by a single user or can be configured on a same computer for use by multiple users. For single user the scripts can be resided in the user's home directory where he can make the program execution calls. However for multiple users the scripts have to be located in a directory accessible to all users typically owned by root. The following two environmental variables have to be set specifying the location of the stored scripts.

(i) Include the scripts directory in the PATH and make sure all files in the directory have the execute permissions. For example ..
    export PATH=$PATH:/usr/local/pkg/snp_phage/Software/scripts
(ii) Set the environmental variable for SNP_PHAGE_LIB. For example
    export SNP_PHAGE_LIB=/usr/local/pkg/snp_phage/Software/scripts/lib

The Config directory has 4 files that needs to be modified.

(i) myDatabase.pm for specifying database connections as discussed above
(ii) myDirectories.pm for specifying the file locations that are processed through the pipeline. During testing these can be set to match the directories in the test_data and can be later changed as per the production needs. The following directory locations have to be specified. Also check for file permissions to these directories.

The first 3 directories are part of the snp-phage software package.

-- scriptsDir (location where the snp-phage scripts are located
-- libDir   (perl modules within the snp-phage)
-- configDir (this directory contains this script and myDatabase.pm)

These can be initially configured to match the directory names in the test_data folder during testing and subsequently new directories can be created and specified to meet the individual needs.

-- batchSeqIDsDir (To store file containing list of SeqIDs / or current directory i.e., ./)
-- temp_dir_path (To store the files created when the programs are being executed)
-- store_dir_path (The required files from temp directory above are copied here)
-- ChromatsDirBeforeRenaming
-- ChromatsDirAfterRenaming

(iii) myExecutables.pm This script has not been fully configured with the understanding that all executables are set in the PATH and their full path need not have to be specified. However, for specific instances where such provision is not applicable this script can be used to store the path of executables in a one common location for access by all scripts.

(iv) myProgramOptions.pm This script contains the program options for the scripts executed during a run. The phredPhrap, polybayes and polyphred program options are set using this script and can be modified by a user.

4. Checking installation

A script Software/TestConfig.pl is provided to check for pre-requisites

It makes the following tests:

1. All perl modules specified above are installed
2. Directories specified in myDirectories.pm are valid and the user has write permissions to those directories.
3. Database connections are valid and user can connect to the database and perform queries

You have to independently make sure that the packages phred, phrap, consed, polybayes, polyphred and C4.5 are installed and are in the path to execute directly without specifying the path. Otherwise you have to specify that in the myExecutables.pm and call them when using those programs.

5. Setting up Web Interface

5.a Web Pages

To enhance the user experience with web pages the following were used

- *SSI* (server side includes)
- *CSS* (cascading style sheets) and
- *Javascript*

For enabling the server side includes you have to place the web pages in a directory (for example /var/www/html/snp_phage). It can be specified in the httpd.conf file. The 3 bold lines are important to view the server side includes. After changing the configuration you have to restart the service

```
<Directory "/var/www/html/snp_phage">
  AddHandler server-parsed .shtml
  AddType text/html .shtml
  Options +Includes
  AllowOverride AuthConfig
  Order allow,deny
  Allow from all
</Directory>
```

In the Software/HTML directory all the files required to recreate the demo website were included. You can copy them to a web accessible folder and may later like to customize the content / appearance of the website to suit your specific project needs. The webpage **header.html** contains the left hand side menu and has to be updated with the cgi-bin directory path and relevant links for your project.

5.b CGI-Perl scripts

The perl scripts provided in the cgi-bin directory can be copied into your local cgi-bin directory of choice and the two scripts myDatabase.pm and myDirectories.pm have to be configured.

Specify MySQL database name/username/password for myDatabase.pm and
Specify directory location for the following directories.
(during the test you can use the test database and test data directories)

-- html_local (This is the path eg., /var/www/html/yourdirectory/)
-- html (For access from website eg., /yourdirectory/)
-- cgi_bin (For access from website /cgi-bin/yourdirectory/)
-- store_dir_path
(This should be the same path you specified in the Config/myDirectories.pm)
The files under edit_dir have to be made available for public view.

For example: The html directory path from web interface for

http:://www.mysite.edu/todd/ would be /todd/ where as the html_local would be /var/www/html/todd/

5.c Security

The code presented here will create web pages and cgi-bin scripts. There are inherent risks and vulnerabilities that come along with the general problems associated with the different web enabling software. We do not offer any implicit or explicit guarantees about the integrity/vulnerability of site hosting these pages. We recommend keeping the website open to specific internal IP addresses and consult a security analyst for making your data openly available for the world.

6. Renaming chromatogram files

For easy parsing of data it is advisable to have a uniform file naming convention to encode as much details possible about the project. Here we propose a convention containing the basic information necessary to associate a chromatogram with its origin.

The file name has three necessary parts

The filename has three necessary parts that are necessary for identification

(i) STS identifier (6 alpha numeric characters)
(ii) Individual name (7 alpha numeric characters)
(iii) Direction of the read (b or g depending on the direction of the read)

For example for the chromatogram
P13_035397JaloEEP.b_01.ab1

035397 is the STS identification number
JaloEEP is the identifier name and
b represents the direction of the read.

The values P13 and 01 are the details about to the well location and lane number of the sequencing run and are not used by the program further.

P13 represents the well location on the sequencing plate (optional) and
01 is the lane number of the sequencer capillary (optional).

The package includes a Perl script ***renameFiles.pl*** that takes two arguments

1. A tab delimited file containing 4 fields for each column that are (current file name and STS identifier, individual name and direction of the read)
2. Option to copy /move files into a new folder after renaming.
3. Option to save old/new file names to a file

Program usage : renameFiles.pl <FILE_NAME> <copy/move> <save Old/New FileNames yes/no>

<file_name>

The file should contain the following information in the tab delimited format

1. Current File name (along with the path)
2. STS identifier (upto 6 characters in length --rest will be trimmed)
3. Individual Identifier for eg., Breed, Cultivar,Population or Person Name
(upto 7 characters in length --rest will be trimmed)
4. Read Direction (Forward / Reverse)

<copy/move>

copy -- To rename the file and new file is stored in another directory
move -- To move the original file into another directory after renaming

<save Old/New FileNames>

yes -- saves the new and old file names to a file
no -- show the new and old files only on the screen

3 sample files are included in the testdata/ ChromatsDirBeforeRenaming that can be modified by running this script as

perl renameFiles.pl TestRename.txt copy yes

with names not following the convention. A file TestRename.txt containing the old file name and the

7. Processing Files

If the scripts are all configured correctly the software can be tested with the test files by typing.

$ perl batch_process.pl IDFILE.txt

The output will be as follows:

run_phredPhrap.pl 015471

POLYPHRED Version 5.04

Reading the ACE file
/data2/lmatukum/Software/SNP_PHAGE/test_data/TempDataStore/edit_dir/015471.fasta
.screen.ace.1
Reading the PHD and POLY files.
  Reading information for contig Contig1
Processing the contig Contig1
Searching for SNPs
Searching for insertion / deletion sites.
Updating the ACE file
/data2/lmatukum/Software/SNP_PHAGE/test_data/TempDataStore/edit_dir/015471.fasta
.screen.ace.1
Updating the PHD files.
  Updating information for Contig1
Writing to standard output.

Finished 015471
