# Supplementary figures and images for: SNP-PHAGE – High throughput SNP discovery pipeline
Source: BMC Bioinformatics. 2006 Oct 23;7:468. doi: 10.1186/1471-2105-7-468 (PMC1626092; doi:10.1186/1471-2105-7-468)

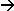

Supplement: Additional file 1 — SNP-PHAGE software package. This compressed file contains all scripts required to create a SNP processing pipeline and a web interface for data analysis and visualization that is powered by a backend relational database. [file 1471-2105-7-468-S1.gz › Software/HTML/Arrow.jpg]

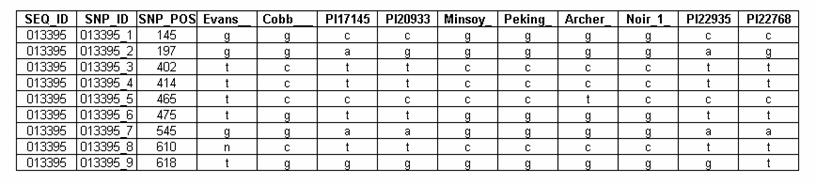

Supplement: Additional file 1 — SNP-PHAGE software package. This compressed file contains all scripts required to create a SNP processing pipeline and a web interface for data analysis and visualization that is powered by a backend relational database. [file 1471-2105-7-468-S1.gz › Software/HTML/CultivarFile.jpg]

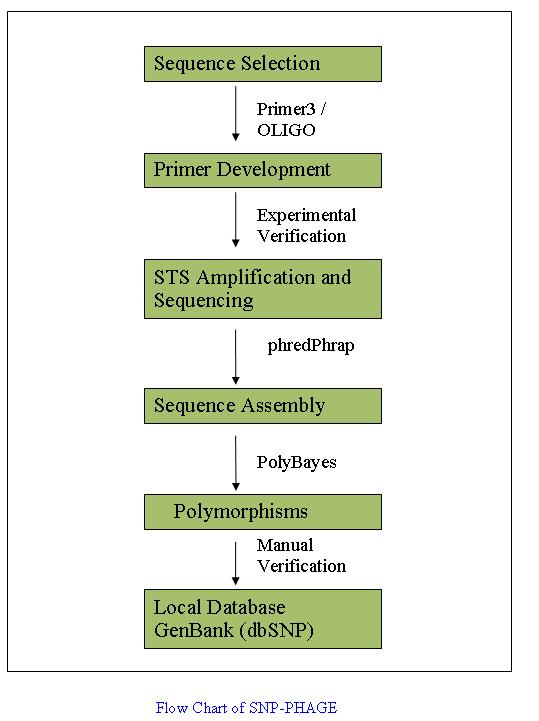

Supplement: Additional file 1 — SNP-PHAGE software package. This compressed file contains all scripts required to create a SNP processing pipeline and a web interface for data analysis and visualization that is powered by a backend relational database. [file 1471-2105-7-468-S1.gz › Software/HTML/FlowChart.jpg]

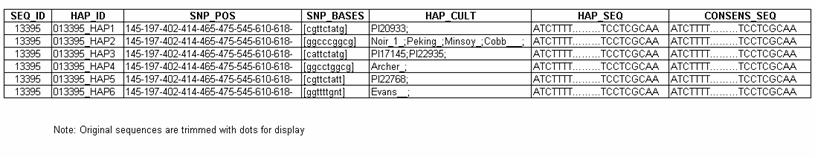

Supplement: Additional file 1 — SNP-PHAGE software package. This compressed file contains all scripts required to create a SNP processing pipeline and a web interface for data analysis and visualization that is powered by a backend relational database. [file 1471-2105-7-468-S1.gz › Software/HTML/HaplotypeFile.jpg]

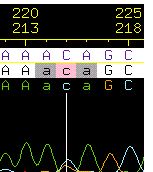

Supplement: Additional file 1 — SNP-PHAGE software package. This compressed file contains all scripts required to create a SNP processing pipeline and a web interface for data analysis and visualization that is powered by a backend relational database. [file 1471-2105-7-468-S1.gz › Software/HTML/Hetero.jpg]

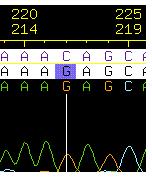

Supplement: Additional file 1 — SNP-PHAGE software package. This compressed file contains all scripts required to create a SNP processing pipeline and a web interface for data analysis and visualization that is powered by a backend relational database. [file 1471-2105-7-468-S1.gz › Software/HTML/Homo1.jpg]

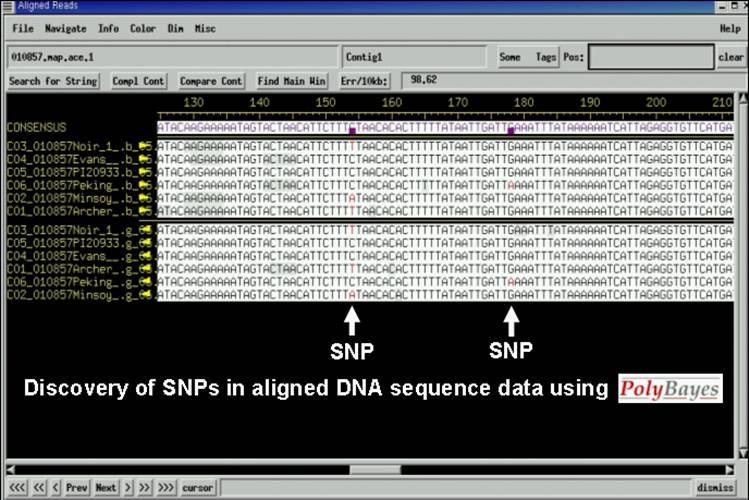

Supplement: Additional file 1 — SNP-PHAGE software package. This compressed file contains all scripts required to create a SNP processing pipeline and a web interface for data analysis and visualization that is powered by a backend relational database. [file 1471-2105-7-468-S1.gz › Software/HTML/Introduction_clip_image002.jpg]

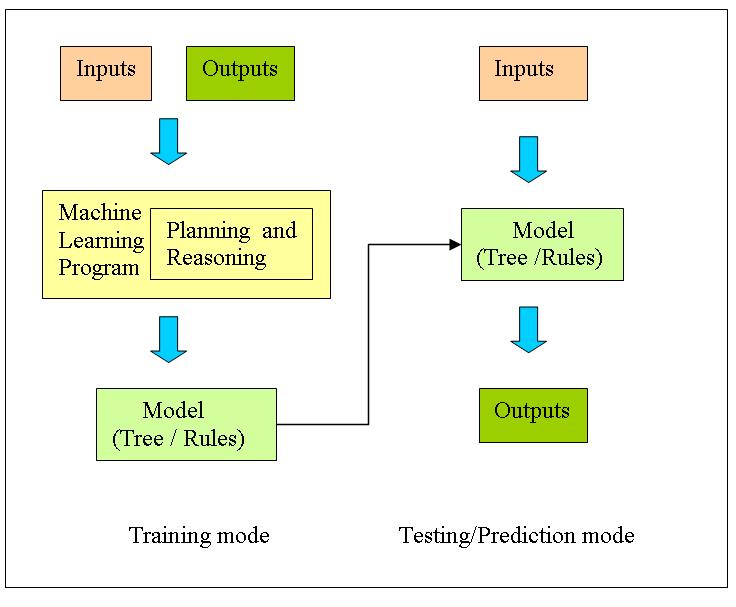

Supplement: Additional file 1 — SNP-PHAGE software package. This compressed file contains all scripts required to create a SNP processing pipeline and a web interface for data analysis and visualization that is powered by a backend relational database. [file 1471-2105-7-468-S1.gz › Software/HTML/ML_General.jpg]

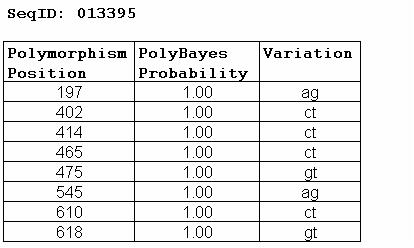

Supplement: Additional file 1 — SNP-PHAGE software package. This compressed file contains all scripts required to create a SNP processing pipeline and a web interface for data analysis and visualization that is powered by a backend relational database. [file 1471-2105-7-468-S1.gz › Software/HTML/SNP_FILE1.jpg]

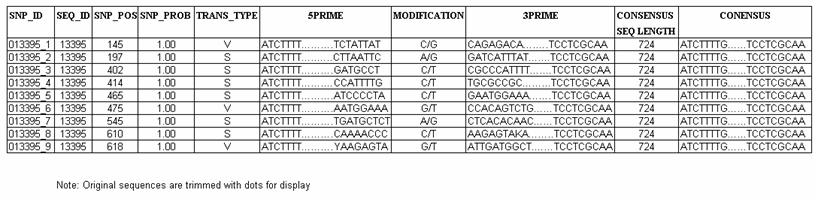

Supplement: Additional file 1 — SNP-PHAGE software package. This compressed file contains all scripts required to create a SNP processing pipeline and a web interface for data analysis and visualization that is powered by a backend relational database. [file 1471-2105-7-468-S1.gz › Software/HTML/SNP_FILE2.jpg]

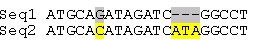

Supplement: Additional file 1 — SNP-PHAGE software package. This compressed file contains all scripts required to create a SNP processing pipeline and a web interface for data analysis and visualization that is powered by a backend relational database. [file 1471-2105-7-468-S1.gz › Software/HTML/SNP.jpg]

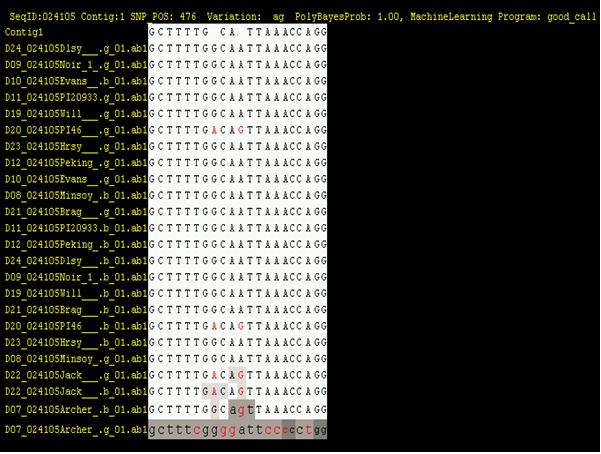

Supplement: Additional file 1 — SNP-PHAGE software package. This compressed file contains all scripts required to create a SNP processing pipeline and a web interface for data analysis and visualization that is powered by a backend relational database. [file 1471-2105-7-468-S1.gz › Software/HTML/SNP_Visual.jpg]

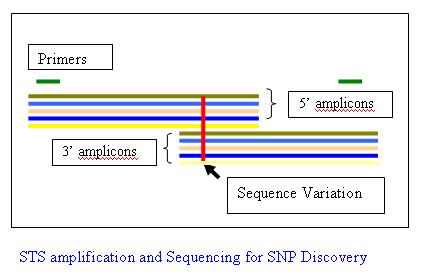

Supplement: Additional file 1 — SNP-PHAGE software package. This compressed file contains all scripts required to create a SNP processing pipeline and a web interface for data analysis and visualization that is powered by a backend relational database. [file 1471-2105-7-468-S1.gz › Software/HTML/STS2.jpg]

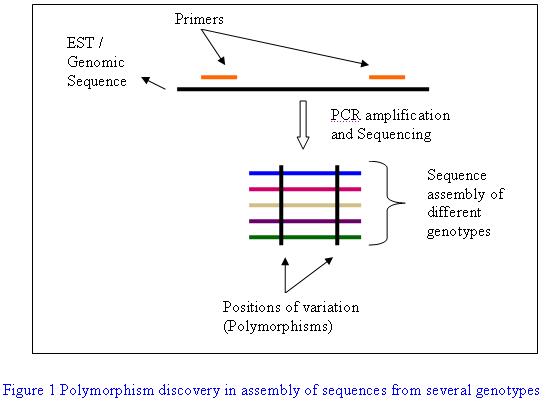

Supplement: Additional file 1 — SNP-PHAGE software package. This compressed file contains all scripts required to create a SNP processing pipeline and a web interface for data analysis and visualization that is powered by a backend relational database. [file 1471-2105-7-468-S1.gz › Software/HTML/STS.jpg]

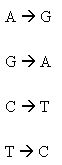

Supplement: Additional file 1 — SNP-PHAGE software package. This compressed file contains all scripts required to create a SNP processing pipeline and a web interface for data analysis and visualization that is powered by a backend relational database. [file 1471-2105-7-468-S1.gz › Software/HTML/Transition.jpg]

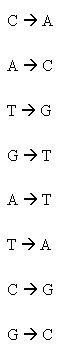

Supplement: Additional file 1 — SNP-PHAGE software package. This compressed file contains all scripts required to create a SNP processing pipeline and a web interface for data analysis and visualization that is powered by a backend relational database. [file 1471-2105-7-468-S1.gz › Software/HTML/Transversion.jpg]

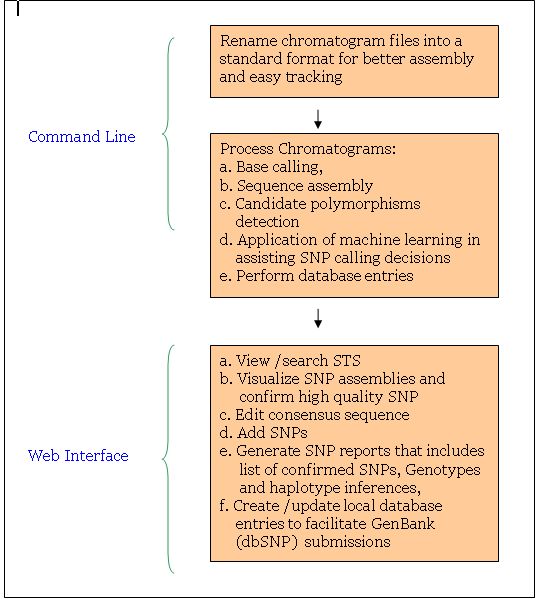

Supplement: Additional file 1 — SNP-PHAGE software package. This compressed file contains all scripts required to create a SNP processing pipeline and a web interface for data analysis and visualization that is powered by a backend relational database. [file 1471-2105-7-468-S1.gz › Software/HTML/FlowChart_SNP_Phage.jpg]
